# Clinical effects of Apatinib combined with DOS neoadjuvant chemotherapy regimen in neoadjuvant chemotherapy for LAGC

**DOI:** 10.12669/pjms.37.7.4265

**Published:** 2021

**Authors:** Peng-zhe Zhou, Lei Gao, Wei Wu, Ying-xia Hao

**Affiliations:** 1 Peng-zhe Zhou, Department of Gastroenterology, Baoding First Central Hospital, Hebei, Baoding, 071000, P. R. China; 2 Lei Gao, Department of Gastroenterology, Baoding First Central Hospital, Hebei, Baoding, 071000, P. R. China; 3 Wei Wu, Department of Gastroenterology, Baoding First Central Hospital, Hebei, Baoding, 071000, P. R. China; 4 Ying-xia Hao Department of Gastroenterology, Baoding First Central Hospital, Hebei, Baoding, 071000, P. R. China

**Keywords:** Apatinib, DOS, LAGC, Neoadjuvant chemotherapy

## Abstract

**Objective:**

To evaluate the clinical effects of apatinib combined with DOS regimen in the neoadjuvant chemotherapy for locally advanced gastric cancer (LAGC).

**Methods:**

Eighty patients with LAGC admitted to Baoding first Central Hospital from January 2018 to October 2020 were randomly divided into two groups (n=40, respectively). The control group received DOS chemotherapy regimen alone. The experiment group additionally orally took apatinib mesylate tablets. The changes in CEA, CA19-9 and other tumor markers, RO resection rate, incidence of operative complications, adverse reactions, and other indicators were compared between the two groups.

**Results:**

The overall response rate (ORR) of the experimental group was 72.5%, which was significantly better than that of the control group (50%) (*p*=0.03). After the treatment, the CEA and CA19-9 in the experiment group were significantly lower than those in the control group (*p*=0.00). The Ro resection rate was 77.5% in the experiment group and 57.5% in the control group (*p*=0.03). The operation time was shortened and amount of bleeding decreased in the experiment group, and the differences were statistically significant (*p*=0.00). The incidence of surgical complications in the experimental group was 17.5%, significantly lower than that in the control group (37.5%) (*p*=0.04).

**Conclusion:**

Apatinib combined with DOS regimen is effective for patients with LAGC without significantly increasing adverse reactions. Meanwhile, tumor markers are reduced significantly. Besides, the Ro resection rate and the incidence of operative complications are obviously superior to the DOS neoadjuvant chemotherapy regimen alone.

## INTRODUCTION

Gastric cancer (GC) is one of the most common malignant tumors of digestive tract.^1^ Its morbidity and mortality rate rank the 2nd among malignant tumors throughout the world. Its early onset is insidious; and the symptoms lack specificity. Therefore, some patients are at the middle and advanced stages when confirmed, losing the optimal treatment period.^2^ GC patients are characterized with low radical operation (Ro) resection rate and high mortality rate.^3^

In recent years, more and more clinical studies have shown that, neoadjuvant chemotherapy can improve the overall survival (OS) of patients, and have proved that the main therapies for advanced GC are neoadjuvant chemoradiotherapy, molecular targeted therapy, immunotherapy and other comprehensive treatment plans that really benefit patients.^4^ Since LAGC has microsatellite instability (MSI) state, patients with LAGC probably cannot benefit from neoadjuvant therapies. Patients with LAGC may benefit more from the combined immunotherapy or targeted therapy.^5^ Some studies have found that the neoadjuvant chemotherapy combined with immunotherapy or targeted therapy can reduce the gross tumor volume (GTV) and increase the chance of surgical removal.^6^ We treated LAGC with apatinib combined with DOS neoadjuvant chemotherapy regimen and achieved some effects as reported below.

## METHODS

One hundred eighty patients with LAGC admitted to Baoding first Central Hospital from January 2018 to October 2020 were selected and randomly divided into two groups (n= 40, respectively). There were 21 male and 19 female patients aged 53~71 (average 65.14±8.47 years) in the experiment group. There were 23 male and 17 female patients aged 51~73 (average 67.05±8.31 years) in the control group. There was no significant difference in the general data of patients between the two groups. However, there still was comparability between the two groups (Table-I).

**Table I:** Contrastive analysis of general data between the experiment group and the control group (X¯±S) n=40.

*Indicator*	*Experiment group*	*Control group*	*t/χ^2^*	*p*
Age (y)	65.14±8.47	67.05±8.31	1.02	0.31
Male (%)	21 (52.5%)	23 (57.5%)	0.20	0.65
***Pathological type***				
Papillary adenocarcinoma (%)	13 (32.5%)	16 (40%)	0.49	0.23
Canalicular adenoma (%)	11 (27.5%)	10 (25%)	0.06	0.78
Mucinous cell carcinoma (%)	7 (17.5%)	6 (15%)	0.09	0.76
Signet-ring cell carcinoma (%)	4 (10%)	2 (5%)	0.72	0.36
Miscellaneous (%)	5 (12.5%)	6 (15%)	0.11	0.74
***Tumor location***				
Whole stomach (%)	2 (5%)	1 (2.5%)	0.35	0.55
Cardia (%)	18 (45%)	20 (50%)	0.20	0.65
Antrum of stomach (%)	9 (22.5%)	6 (15%)	0.74	0.39
Body of stomach (%)	11 (27.5%)	13 (32.5%)	0.24	0.63

P > 0.05.

### Ethical approval

The study was approved by the Institutional Ethics Committee of Baoding First Central Hospital at February 7, 2021, and written informed consent was obtained from all participants.

### Inclusion criteria:

1. Patients diagnosed with GC by imaging examination and gastroscopic histopathological biopsy.^7^
2. Patients with LAGC (stage III) as suggested by CT and other imaging examinations, with lesions that can be accurately measured.
3. Patients with a KPS score ≥ 70 points, and an expected OS ≥ 6 months.
4. Patients whose families were willing and able to cooperate in completing the study and had good treatment compliance.
5. Patients who had no contraindications for the drugs used in this study.

### Exclusion criteria:

1. Patients complicated with severe cardiopulmonary dysfunction.
2. Patients complicated with other malignant tumors.
3. Patients who had mental or cognitive dysfunction and could not complete the study.
4. Patients who had or were complicated with severe complications and could not tolerate operation.
5. Patients who had taken orally relevant drugs that might affect the study, such as immunosuppressor and hormone.

### Therapies

Patients in both groups received blood cell analysis and examination of liver function and kidney function; and abnormal indicators were corrected accordingly. Nutrition assessment was performed during the treatment. Nutrition support therapy was provided for malnourished patients. Patients with corresponding symptoms were given basic treatment, such as antiemetic treatment to correct electrolyte disorder. Hydration was performed the day before chemotherapy.

The control group was given DOS chemotherapy regimen (docetaxel + oxaliplatin + tegafur, gimeracil and oteracil potassium): docetaxel 60mg/m^2^, intravenous drip d1, oxaliplatin 100mg/m^2^, intravenous drip d2, tegafur, gimeracil and oteracil potassium (body surface area < 1.25m^2^, 40mg/time; body surface area 1.25~1.50m^2^, 50mg/time; body surface area > 1.5m^2^, 60mg/time) oral administration bid d1~10; every 21 d is a treatment cycle.^8^ On this basis, the experiment group additionally orally took apatinib mesylate tablets without interruption, 850mg/d. Every 3 weeks is a cycle. Adverse reactions in patients in both groups were evaluated at the end of each treatment cycle. The effects were evaluated at the end of every two treatment cycles. The operative treatment was performed after 3~4 cycles of chemotherapy. All patients underwent laparoscopic surgery. If intraoperative adhesion was serious and it was difficult to perform endoscopic surgery, open surgery should be performed. All patients were followed up for 6 months after the operation.

### Observation indicators:

### 1. Effect evaluation:

The effects in all patients were evaluated at the end of every two treatment cycles. The tumor was evaluated according to the Response Evaluation Criteria in Solid Tumors 1.0 (RECIST 1.0):^9^ complete response (CR): the lesion disappeared completely; partial response (PR): the total measured diameters of the target lesions were reduced by 30% from the baseline; stable disease (SD): the longest diameter of the lesion was reduced by 25%~50%; progression disease (PD): the total long diameters of all target lesions increased by at least 20%, and the absolute value of the increase in the total long diameters was greater than 5mm; or new lesions appeared. Overall response rate (ORR) = (CR+PR)/total number×100%.

### 2. Evaluation of adverse drug reactions (ADRs):

The ADRs after one treatment cycle in both groups were recorded, including bone marrow suppression, gastrointestinal reactions, peripheral neuritis and liver function injury.

### 3. Contrastive analysis of tumor markers

Morning fasting blood was sampled before and after the treatment to detect carcino-embryonic antigen (CEA), CA19-9 and other tumor markers; and the differences between the two groups were compared and analyzed.

### 4. Evaluation of operation indicators

The Ro resection rate and the incidence of operative complications in the two groups were evaluated. The operative complications were graded in accordance with the Clavien grading system:^10^

**Grade-I:** Complications requiring no drug or operation, endoscope and other interventions, drugs include antiemetics, antipyretics, pain relievers, diuretics, electrolyte supplements, and physical therapies; Grade-I also includes the complications in patients whose stitches were taken out without other treatment at the bedside due to incision infection.

**Grade-II:** Complications requiring drug therapies, including blood transfusion or total parenteral nutrition (TPN).

**Grade-III:** Complications requiring surgical, endoscopic, or radiotherapeutic interventions.

**Grade-IV:** life-threatening complications (including central nervous system complications, CNSCs) requiring treatment in the intensive care unit (ICU).

**Grade-V:** Death.

### Statistical analysis:

The software SPSS 20.0 was used for the statistical analysis of all data. The measurement data were expressed as (X¯±S). Independent samples t-test was used for the data analysis between the two groups. Paired t test was applied to intra-group data analysis. χ^2^ tests were used for rate comparison. A *p*-value of <0.05 was considered statistically significant.

## RESULTS

The contrast analysis of the effects between the two groups is shown in Table-II. It suggested that the ORR was 72.5% in the experiment group and 50% in the control group. The effects in the experiment group were evidently better than those in the control group, and the difference was statistically significant (*p*=0.03).

**Table II:** Contrastive analysis of effects between two groups (X¯±S) n=40.

*Group*	*CR*	*PR*	*SD*	*PD*	*ORR*
Experiment group	7	22	8	3	29 (72.5%)
Control group	3	17	14	6	20 (50%)
c^2^					4.27
p					0.03

p< 0.05.

The contrast analysis of the incidence of ADRs after the treatment between the two groups suggested that the incidence of adverse reactions was 50% in the experiment group and 40% in the control group. Although the incidence of adverse reactions in the experiment group was higher than that in the control group, the difference was not statistically significant (*p*=0.36). (Table-III)

**Table III:** Contrastive Analysis of ADRs after Treatment Between Two Groups (X¯±S) n=40.

*Group*	*Bone marrow suppression*	*Gastrointestinal reactions*	*Peripheral neuritis*	*Liver function injury*	*Incidence*
Experiment group	4	7	3	6	20 (50%)
Control group	5	4	2	5	16 (40%)
c^2^					0.81
p					0.36

p< 0.05.

There was no significant difference in the pre-treatment levels of CEA and CA19-9 between the two groups (*p*> 0.05). These indicators decreased after the treatment, and the difference was statistically significant (*p*< 0.05). The post-treatment levels of CEA and CA19-9 in the experiment group were significantly lower than those in the control group, and the difference was statistically significant (*p*= 0.00) (Table-IV).

**Table IV:** Contrastive analysis of tumor marker levels before and after treatment between two groups (X¯±S) n=40.

*Group*	*CEA (ng/mL)*				*CA19-9 (U/mL)*			

	Before the treatment*	*After the treatmentΔ*	*t*	*p*	Before the treatment*	*After the treatmentΔ*	*t*	*p*
Experiment groupΔ	46.47±8.32	26.04±8.63	10.78	0.00	53.20±8.65	36.58±6.70	9.61	0.00
Control groupΔ	46.36±8.54	32.57±8.42	7.27	0.00	53.19±8.81	45.63±5.83	4.53	0.00
t	0.06	3.43			0.01	6.44		
p	0.95	0.01			0.10	0.00		

* p> 0.05, Δ*p*< 0.05.

The contrastive analysis of operation indicators between the two groups suggested that the Ro resection rate was 77.5% in the experiment group and 57.5% in the control group, showing statistically significant difference (*p*= 0.03). Moreover, the experiment group had some advantages over the control group in terms of the operation time and the amount of bleeding: the operation time was shortened and amount of bleeding decreased in the experiment group, and the differences were statistically significant (*p*= 0.00). The post-operative complications in the two groups were mild (Clavien grades I or II). No severe complications occurred. The incidence of operative complications was 17.5% in the experiment group and 37.5% in the control group; and the incidence of complications in the experiment group was significantly lower than that in the control group (*p*=0.04) (Table-V).

**Table V:** Comparative analysis of operation conditions between two groups ( ±S) n=40.

*Indicator*	*Experiment group*	*Control group*	*t/χ^2^*	*p*
Ro resection rate (%) *	31 (77.5%)	23 (57.5%)	4.71	0.03
Operation time (min) *	147.37±34.52	186.47±36.85	4.88	0.00
Amount of bleeding (ml) *	79.62±13.49	113.85±23.71	7.94	0.00
Operative complications (Clavien %) *	7 (17.5%)	15 (37.5%)	4.01	0.04
Grade-I	4	8	
Grade-II	3	7	

* p < 0.05.

## DISCUSSION

The standard therapies for LAGC are different around the world. In Western countries, perioperative chemotherapy or postoperative adjuvant chemoradiotherapy is the preferred therapy. In Asia, however, gastrectomy after neoadjuvant chemotherapy remains the standard therapy. The addition of targeted drugs to cytotoxic chemotherapy also seems promising as a standard therapy for LAGC.^11^ The study of Ma et al.^12^ confirmed that neoadjuvant chemotherapy has certain benefits for prolonging the long-term survival rate of patients with LAGC and increasing the Ro resection rate and has certain safety.

In the study of Coccolini et al.,^13^ the treatment of LAGC with operation alone and operative treatment after neoadjuvant chemotherapy were compared. The results showed that the 5-year survival rate and Ro resection rate in patients underwent operative treatment after neoadjuvant chemotherapy were significantly higher than those in patients receiving operative treatment alone, and the differences were statistically significant. The study of Li et al.^14^ showed that patients with LAGC who had received neoadjuvant chemotherapy had higher safety and operation tolerance. Nevertheless, according to the study of Bauer et al.,^15^ neoadjuvant chemotherapy during perioperative period is reasonable and independent of tumor location or age. Chemotherapy can significantly lower the serum levels of CEA and CA 125 in patients with GC, and reduce the chance of local postoperative recurrence and distant metastasis.^16^

The DOS regimen includes docetaxel, oxaliplatin, and tegafur, gimeracil and oteracil potassium. Docetaxel, a semisynthetic taxane antitumor drug, can significantly prolong the survival time and increase the remission rate in patients with advanced GC.^17^ The study of Biffi et al.^18^ showed that, treating the patients with LAGC with the neoadjuvant chemotherapy based on docetaxel before the operation can effectively improve the Ro resection rate. Oxaliplatin is a new platinum antitumor drug, and such advantages as broad spectrum, low toxicity and high-water solubility. Its toxicity is low, causing no severe gastrointestinal reaction. Moreover, it is also effective for other platinum-resistant patients.^19^ Tegafur, gimeracil and oteracil potassium is a compound preparation of a new generation of fluorouracil. The study of Liu et al.^20^ showed that the oxaliplatin and tegafur, gimeracil and oteracil potassium regimen has such advantages as high efficiency, low toxicity and good tolerance.

Apatinib is a new small molecule selective vascular endothelial growth factor receptor 2 (VEGFR-2) tyrosine kinase inhibitor, and is effective for advanced gastric adenocarcinoma, non-small cell lung cancer (NSCLC), breast cancer, gynecological cancer, hepatocellular carcinoma (HCC), thyroid cancer and sarcoma. Apatinib significantly prolongs the progression-free survival (PFS) and OS, and has certain effects for patients with advanced GC or with progression or recurrence after chemotherapy when used in combination with other chemotherapy drugs.^21^ Compared with placebo, apatinib has single-agent activity, and has the advantage that chemotherapy drugs cannot compare, especially for tumor patients with high MSI.^22^ The study of Yang et al.^23^ showed that the treatment with apatinib can relieve certain clinical symptoms, but it can’t significantly improve the quality of life (QOL).

The adverse reactions of apatinib combined with chemotherapy drugs, mainly neuritis, are acceptable and controllable.^24^ The study of Cheng et al.^25^ suggested that the main adverse reactions are hypertension, albuminuria and hand-foot syndrome (HFS). However, Geng et al.^26^ held that the adverse reactions of anti-angiogenic drugs may indicate that these drugs are effective.

This study showed that the ORR of apatinib combined with DOS neoadjuvant chemotherapy regimen was 72.5%, which was much higher than that of the chemotherapy alone group (*p*=0.03); while the difference in the incidence of adverse reactions between the two groups was not statistically significant (*p*=0.36). After the treatment, the CEA and CA19-9 in the experiment group were significantly lower than those in the control group, showing statistically significant difference (*p*=0.00). The Ro resection rate was 77.5%, which was significantly higher than that in the control group (*p*=0.03). In addition, the operation time was shortened and amount of bleeding decreased in the experiment group (*p*=0.00). The incidence of operative complications was lower than that in the control group (17.5%: 37.5%, *p*=0.04).

### Limitations of the study

The shortcomings of this study include small sample size, short follow-up time, and failure to divide and study the post-operative pathological types, therapeutic effects and prognosis of patients in a more detailed manner due to small sample size. We are actively increasing the sample size and further prolonging the follow-up time. Besides, we are further detailing the study, in order to make more objective evaluations of the influence of this therapy in different pathological types and its long-term effects.

## CONCLUSION

In conclusion, apatinib combined with DOS regimen is effective for patients with LAGC without significantly increasing adverse reactions. Meanwhile, tumor markers are reduced significantly. Besides, the Ro resection rate and the incidence of operative complications are obviously superior to the DOS neoadjuvant chemotherapy regimen alone.

### Authors’ Contributions:

**PZ** **and** **YH** designed this study and prepared this manuscript, and are responsible and accountable for the accuracy or integrity of the work.

**LG** collected and analyzed clinical data.

**WW** significantly revised this manuscript.
